# Environmental and Air Pollution Factors Affecting Allergic Eye Disease in Children and Adolescents in India

**DOI:** 10.3390/ijerph18115611

**Published:** 2021-05-24

**Authors:** Anthony Vipin Das, Sayan Basu

**Affiliations:** 1 Department of eyeSmart EMR & AEye, L V Prasad Eye Institute, Hyderabad 500034, India; vipin@lvpei.org; 2 The Cornea Institute, L V Prasad Eye Institute, Hyderabad 500034, India; 3 Centre for Ocular Regeneration (CORE), L V Prasad Eye Institute, Hyderabad 500034, India; 4 Prof. Brien Holden Eye Research Centre (BHERC), L V Prasad Eye Institute, Hyderabad 500034, India

**Keywords:** allergic eye disease, trend analysis, environmental pollution

## Abstract

The aim of this study was to describe the correlation between the meteorological and air pollution parameters with the temporal pattern of presentation of recent onset allergic eye disease (AED). This cross-sectional hospital-based study included new patients (≤21 years of age) presenting between January 2016 and August 2018 from the district of Hyderabad with a clinical diagnosis of AED and an acute exacerbation of recent onset of symptoms of less than 3 months duration. Correlation analysis was performed with the local environmental rainfall, temperature, humidity, windspeed, and air pollution. Of the 25,354 new patients hailing from the district of Hyderabad, 2494 (9.84%) patients were diagnosed with AED, of which 1062 (4.19%) patients had recent onset of symptoms. The mean monthly prevalence in this cohort was 4.13%, and the month of May (6.09%) showed the highest levels. The environmental parameters of humidity (r^2^ = 0.83/*p* = < 0.0001) and rainfall (r^2^ = 0.41/*p* = 0.0232) showed significant negative correlation, while temperature (r^2^ = 0.43/*p* = 0.0206) and ground-level ozone (r^2^ = 0.41/*p* = 0.0005) showed significant positive correlation with the temporal pattern of AED in the population. An increase in rainfall and humidity was associated with a lower prevalence, and an increase of temperature and ground-level ozone was associated with a higher prevalence of AED cases during the year among children and adolescents.

## 1. Introduction

The air we breathe is one of the most significant determinants of health of the population. Globally, 9 out of 10 individuals now breathe polluted air that claims over 7 million lives a year [1]. Air pollution poses a serious threat to individuals and is responsible for over one-third of deaths due to chronic obstructive lung disease, lung cancer, stroke, and cardiovascular disease [2]. The main pollutants that affect the mucosal surfaces are the particulate matter (PM), nitrogen dioxide, sulfur dioxide, and ozone at ground level that arise from various sources such as fuel combustion, road traffic, burning fossil fuels, or indoor gas cookers. While the respiratory system majorly takes the brunt of the damage, the ocular surface also is directly exposed to various external environmental factors including air pollution. The irritants in the external environment cause ocular surface inflammation that leads to irritation, watering, burning sensation, and itching impairing the quality of life of the individual. There are many studies that have investigated the relationship between air quality and systemic disease, but there is limited literature on its effect on ocular diseases such as conjunctivitis [3], allergic eye disease [4], and dry eye [5]. Studies have shown that there are subclinical ocular surface changes and a higher level of ophthalmic symptoms in the population exposed to high levels of air pollution [6]. The authors have previously described the demographics, clinical features, and the temporal pattern of the presentation of allergic eye disease in children and adolescents in India, with the trend gradually increasing from the onset of winter and peaking during the summer [7]. In this context, the authors wished to investigate the relationship of the meteorological factors and air pollution indicators on the prevalence of recent onset allergic eye disease in children and adolescents presenting to a multi-tier ophthalmology network in India using electronic medical record-driven analytics.

## 2. Materials and Methods

### 2.1. Study Design, Period, Location, and Approval

This cross-sectional observational hospital-based study included all new patients (≤21 years of age) from the district of Hyderabad presenting between January 2016 and August 2018 to an ophthalmology network located in 200 different geographical locations spread across 4 states (Telangana, Andhra Pradesh, Odisha, and Karnataka) of India [8]. The patient or the parents or guardians of the patient filled out a standard consent form for electronic data privacy at the time of registration. None of the identifiable parameters of the patient information were used for analysis of the data. The study adhered to the Declaration of Helsinki and was approved by the Institutional Ethics Committee. The clinical data of each patient who underwent a comprehensive ophthalmic examination were entered into a browser-based electronic medical records system (eyeSmart EMR) by uniformly trained ophthalmic personnel and supervised by an ophthalmologist using a standardized template [9].

### 2.2. Cases

A total of 25,354 new patients (≤21 years of age) presented to the tertiary and secondary centers of the network during the study period. The inclusion criteria used included the clinical signs of allergic eye disease (AED) as in an earlier publication by the same authors [7]. A total of 2494 patients presented with a clinical diagnosis of AED, of which 1062 patients with an acute exacerbation of recent onset of symptoms of less than 3 months duration were labelled as cases. These cases were included for the trend analysis with the meteorological and pollution parameters obtained for the district of Hyderabad.

### 2.3. Data Retrieval and Processing

The data of 1062 patients included in this study were retrieved from the eyeSmart EMR database system and collated in a single excel sheet. The data on demographics, clinical presentation, and ocular diagnosis of the patients were imported into columns for analysis. Appropriate statistical software was used for analysis of the data that was extracted into the Excel sheet. The weather parameters of 33 districts of the state of Telangana were obtained from the Telangana State Development and Planning Society (TSDPS), Government of Telangana [10]. The pollution parameters of Hyderabad of particulate matter (PM_10_ and PM_2.5_) μg/m^3^, carbon monoxide (CO) mg/m^3^, nitrogen dioxide (NO_2_) μg/m^3^, sulfur dioxide (SO_2_) μg/m^3^, and ozone (O_3_) μg/m^3^ were obtained from the Central Pollution Control Board, Ministry of Environment, Forest and Climate Change, Government of India [11]. The weather and pollution parameters were then compared to the presentation of AED patients from the district of Hyderabad.

### 2.4. Statistical Analysis

The descriptive statistics describing the mean ± standard deviation and median with inter-quartile range (IQR) were used for the demographic data. Prevalence was calculated by dividing the number of AED cases by the total number of patients presenting to the network from the given geographic location during the study period. Correlation coefficient analysis between the prevalence and individual weather parameters was performed using the Medcal software (version 19.2.0). Multiple linear regression analysis was performed using stepwise modeling based on the Akaike information criterion (AIC). The r-squared (r^2^) value of 0.12 or below indicates low, between 0.13 to 0.25 indicates medium, and 0.26 or above indicates high effect size [12].

## 3. Results

### 3.1. Demographics

**Figure 1:**
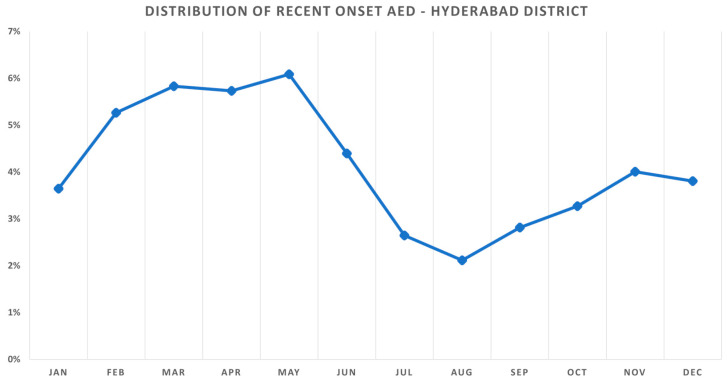
Seasonal variation of recent onset allergic eye disease (AED) in the district of Hyderabad. The prevalence of AED increased steadily from February (5.27%) during winter to peak in the month of May (6.09%) before decreasing with the onset of monsoon in July (2.64%).

Of the 25,354 new patients presenting from the district of Hyderabad during January 2016 to August 2018, 2494 (9.84%) patients were diagnosed with AED and 1062 (4.19%) patients had a recent onset (less than 3 months duration) of the symptoms. The mean age of the patients with AED was 9.82 ± 5.09 years, while the median age was 9 (IQR: 6–13) years. A significant proportion were 730 (68.74%) male patients, and the rest were 332 (31.26%) female patients with AED. The highest prevalence was seen in the month of May with 6.09% (139/2283), and the lowest was seen in the month of August with 2.12% (43/2031). The weather parameters of rainfall, temperature, humidity, windspeed, and the pollution parameters of PM_10_, PM_2.5_, CO, NO_2_, SO_2_, and O_3_ were assessed on a monthly basis. The month-wise distribution of the prevalence of recent onset cases of AED from the district of Hyderabad is shown in Figure 1.

### 3.2. Humidity

**Figure 2:**
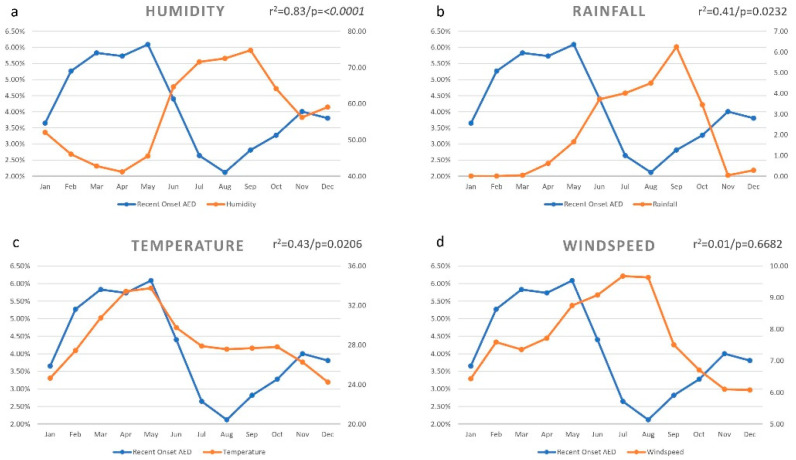
Monthly variation of environmental factors and the prevalence of recent onset allergic eye disease (AED) in the district of Hyderabad. The month-wise prevalence of AED was negatively correlated with the distribution of humidity ((**a**) r = −0.9148) and rainfall ((**b**) r = −0.6462), and positively correlated with temperature ((**c**) r = 0.6558) but not with windspeed ((**d**) r = −0.1383).

The relative humidity distribution per month is shown in Figure 2a. The average relative humidity was 57.56% during 2016–2018. The month of September (74.79%) recorded the highest relative humidity, and the month of April (41.19%), the lowest. There was a negative correlation with the distribution of humidity (r = −0.9148) and the month-wise prevalence of recent onset AED, and this correlation was statistically significant (r^2^ = 0.83/*p* = <0.0001). 

### 3.3. Rainfall

The rainfall distribution per month is shown in Figure 2b. The average rainfall was 2.05 mm/day during 2016–2018. The month of September (6.25 mm/day) recorded the highest rainfall, and the month of February (0.00 mm/day), the lowest. There was a negative correlation with the distribution of rainfall (r = −0.6462) and the month-wise prevalence of recent onset AED, and this correlation was statistically significant (r^2^ = 0.41/*p* = 0.0232).

### 3.4. Temperature

The temperature distribution per month is shown in Figure 2c. The average temperature was 28.43 °C during 2016–2018. The month of May (33.75 °C) recorded the highest average temperature, and the month of December (24.24 °C), the lowest. There was a positive correlation with the distribution of temperature (r = 0.6558) and the month-wise prevalence of recent onset AED, and this correlation was statistically significant (r^2^ = 0.43/*p* = 0.0206).

### 3.5. Windspeed

The windspeed distribution per month is shown in Figure 2d. The average windspeed was 7.72 km/h during 2016–2018. The month of July (9.68 km/h) recorded the highest windspeed, and the month of December (6.07 km/h), the lowest. There was no correlation with the distribution of windspeed (r = −0.1383) and the month-wise prevalence of recent onset AED, and this correlation was not statistically significant (r^2^ = 0.01/*p* = 0.6682).

### 3.6. Particulate Matter 10 (PM_10_)

**Figure 3:**
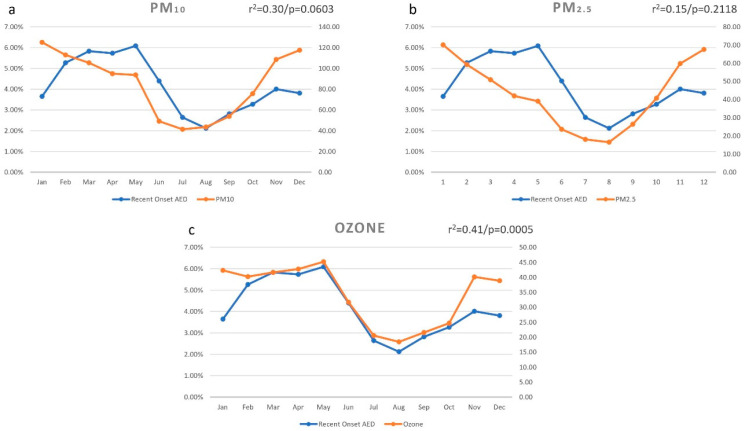
Monthly variation of particulate matter (PM_10_ and PM_2.5_) and ozone on the prevalence of recent onset allergic eye disease (AED) in the district of Hyderabad. The month-wise prevalence of AED was positively correlated with the distribution of PM_10_ ((**a**) r = 0.5563), PM_2.5_ ((**b**) r = 0.3887), and ozone ((**c**) r = 0.8461), which was statistically significant.

The particulate matter distribution (PM_10_) per month is shown in Figure 3a. The average particulate matter reading was 85.88 μg/m^3^ during 2016–2019. The highest particulate matter reading was seen in the month of January (125.09 μg/m^3^), and the lowest in the month of July (41.35 μg/m^3^). There was a positive correlation with the distribution of particulate matter (r = 0.5563) and the month-wise prevalence of recent onset AED, and this correlation was not statistically significant (r^2^ = 0.30/*p* = 0.0603).

### 3.7. Particulate Matter 2.5 (PM_2.5_)

The particulate matter distribution (PM_2.5_) per month is shown in Figure 3b. The average particulate matter reading was 43.46 μg/m^3^ during 2016–2019. The highest particulate matter reading was seen in the month of January (70.08 μg/m^3^), and the lowest in the month of August (16.55 μg/m^3^). There was a positive correlation with the distribution of particulate matter (r = 0.3887) and the month-wise prevalence of recent onset AED, and this correlation was not statistically significant (r^2^ = 0.15/*p* = 0.2118).

### 3.8. Ozone (O_3_)

The ozone at ground level distribution per month is shown in Figure 3c. The average ozone reading was 34.09 μg/m^3^ during 2016–2019. The highest ozone reading was seen in the month of May (45.15 μg/m^3^), and the lowest in the month of August (18.43 μg/m^3^). There was a positive correlation with the distribution of ozone (r = 0.8461) and the month wise prevalence of recent onset AED, and this correlation was statistically significant (r^2^ = 0.41/*p* = 0.0005).

### 3.9. Carbon Monoxide (CO)

**Figure 4:**
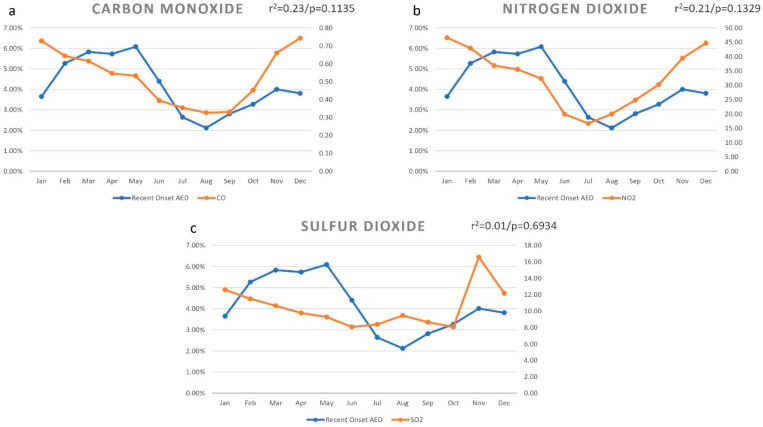
Monthly variation of air pollution indicators and the prevalence of recent onset allergic eye disease (AED) in the district of Hyderabad. The month-wise prevalence of AED was positively correlated with the distribution of carbon monoxide ((**a**) r = 0.4809), nitrogen dioxide ((**b**) r = 0.4595), and sulfur dioxide ((**c**) r = 0.1273), and all were not statistically significant.

The carbon monoxide distribution per month is shown in Figure 4a. The average carbon monoxide reading was 0.53 mg/m^3^ during 2016–2019. The highest carbon monoxide reading was seen in the month of December (0.74 mg/m^3^), and the lowest in the month of August (0.33 mg/m^3^). There was a positive correlation with the distribution of carbon monoxide (r = 0.4809) and the month-wise prevalence of recent onset AED, and this correlation was not statistically significant (r^2^ = 0.23/*p* = 0.1135).

### 3.10. Nitrogen Dioxide (NO_2_)

The nitrogen dioxide distribution as per month is shown in Figure 4b. The average nitrogen dioxide reading was 32.79 μg/m^3^ during 2016–2019. The highest nitrogen dioxide reading was seen in the month of January (46.56 μg/m^3^), and the lowest in the month of July (16.67 μg/m^3^). There was a positive correlation with the distribution of nitrogen dioxide (r = 0.4595) and the month-wise prevalence of recent onset AED, and this correlation was not statistically significant (r^2^ = 0.21/*p* = 0.1329).

### 3.11. Sulfur Dioxide (SO_2_)

The sulfur dioxide distribution per month is shown in Figure 4c. The average sulfur dioxide reading was 10.53 μg/m^3^ during 2016–2019. The highest sulfur dioxide reading was seen in the month of November (16.58 μg/m^3^), and the lowest in the month of October (8.05 μg/m^3^). There was a positive correlation with the distribution of sulfur dioxide (r = 0.1273) and the month-wise prevalence of recent onset AED, and this correlation was not statistically significant (r^2^ = 0.01/*p* = 0.6934).

### 3.12. Multivariate Analysis

**Figure 5:**
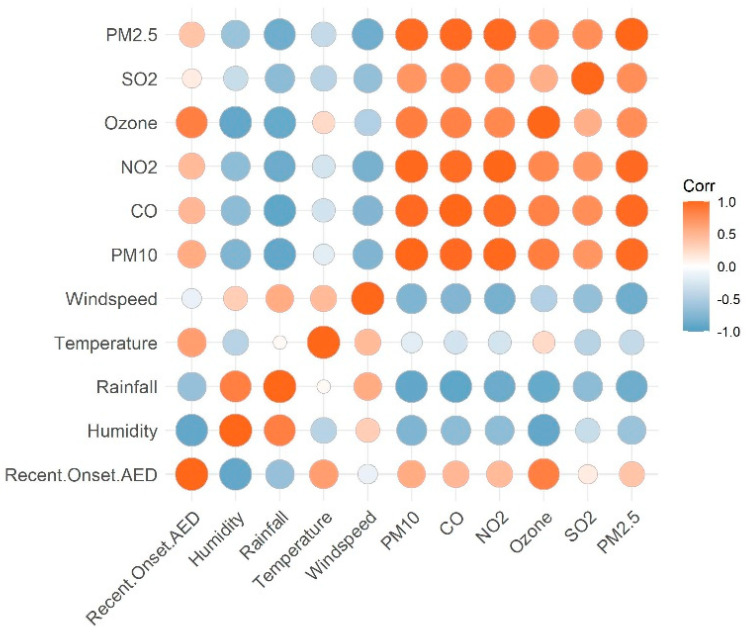
Correlation matrix of environmental factors and air pollution indicators on the prevalence of recent onset allergic eye disease (AED) in the district of Hyderabad.

The environmental variables of relative humidity (r = −0.9204/r^2^ = 0.84/*p* = <0.0001) and temperature (r = −0.6827/r^2^ = 0.46/*p* = <0.0206) were found to be statistically significant on multivariate analysis among all the variables. A correlation matrix of environmental factors and air pollution indicators on the prevalence of recent onset allergic eye disease (AED) is shown in Figure 5.

## 4. Discussion

This study sought to describe the correlation between the meteorological parameters such as environmental temperature, rainfall, humidity, windspeed, and pollution with the temporal pattern of presentation of recent onset allergic eye disease (AED) in a cohort of patients presenting to a multi-tier hospital network in India using electronic medical record-driven analytics. The findings of this study suggest that there is a clear pattern of certain environmental factors with the temporal pattern of AED in the population of the district of Hyderabad, with a peak prevalence seen in the month of May and the lowest seen in the month of August. An increase in humidity and rainfall contributes to a lower prevalence of AED cases during the year, whereas an increase in temperature and ozone at ground level conversely contributes to a higher prevalence of AED cases.

Hong et al. [13] described the effects of ambient air pollution, weather changes, and their effect on outpatient visits for allergic conjunctivitis in Shanghai, China. They found that allergic conjunctivitis was significantly correlated with the levels of NO_2_, ozone, and temperature. The association between humidity and outpatient visits for allergic conjunctivitis was statistically marginal. They also found that the susceptibility to all the above four parameters in patients younger than 40 years of age, while those older than 40 were only consistently correlated with NO_2_. The results are quite similar to our current study as temperature and relative humidity were statistically significant on multivariate analysis among all the variables. The month-wise prevalence of recent onset AED was positively correlated and statistically significant with the distribution of ozone at ground level. However, the AED prevalence was positively correlated with the distribution of nitrogen dioxide, and this correlation was not statistically significant.

Sugiyama et al. [14] studied the effect of PM_2.5_ exposure as an aggravating factor for allergic and respiratory diseases in 2317 school children from the city of Fukuoka. The mean prevalence of ocular symptoms was 11.2% among them. Among the six sources that contributed to the PM_2.5_ in the city, “soil” had a significant positive association with ocular symptoms. Tang et al. [15] described a murine model of acute allergic conjunctivitis induced by continuous exposure to particulate matter (PM_2.5_). The mice that were challenged with eye drops containing PM_2.5_ in increasing concentrations displayed more eyelid edema, tearing, and scratching behavior, and also had a higher goblet cell density in the upper palpebral conjunctiva, showing extensive eosinophil infiltration in the meibomian glands and ocular surface as well. In our study, a positive correlation was found between the levels of PM_10_ and PM_2.5_ with the prevalence of AED cases but was not statistically significant.

Lee et al. [16] described the effect of ozone exposure in a mouse model of allergic conjunctivitis. Two different concentrations of ozone (0.5 and 2.0 ppm) were used, and the conjunctival chemosis and conjunctival injections were significantly higher in the exposed group. They also reported a decrease in the total tear volume after 2 weeks of exposure as compared to the control group. They reported that ozone exposure to the conjunctival epithelial cells led to additional increases in interleukin-6 and tumor necrosis factor-α mRNA levels, which were already induced by treatment with IL-1α. We found a strong positive correlation with the distribution of ozone at ground level and the prevalence of AED cases through the year. This correlation was statistically significant as well, indicating the irritant effects of ozone on the ocular surface, leading to allergic conjunctivitis.

Of all the environmental variables in the study, relative humidity and temperature had the strongest correlation with AED prevalence on multivariate analysis. This reflected the effect of these environmental factors on the possible exacerbation of the disease, which leads to the patients presenting to the hospital to seek care. In India, seasonal variation is distinct, and the increasing pattern of presentation of the AED patients correlates well with the peak of summer and the increasing temperature and decreases with the onset of monsoon with the increasing humidity. The school summer holidays for the children in India are during the months of March to May, and there is an increased exposure to the external environment due to increased outdoor play time of the children. Contrary to what we might possibly expect to see a correlation with air pollution, we did not find any statistical significance with the parameters of particulate matter (PM_10_ and PM_2.5_), nitrogen dioxide, sulfur dioxide, and carbon monoxide, except ground-level ozone with the prevalence of AED cases. Knowledge about this insight can help to better predict the waxing and waning of the chronic nature of the disease and also aid in the management of the expectations of the patients due to its potential to affect their quality of life. There is a need to address this issue at multiples levels of prevention. Primordial prevention constitutes the promotion of clean air, clean energy, and the effects of air pollution on climate change at the policy level; primary prevention helps to identify the vulnerable age groups at risk in the population and sensitize them; secondary prevention involves implementation of prophylactic measures during the early stage of the disease; and tertiary prevention involves treatment of the AED with regular follow up as it is a chronic condition.

The greatest strengths of the study are its large sample size, the longitudinal nature of the cohort, and the use of digital extraction of the medical records for analysis that minimizes human error. The environmental data are sourced from publicly available datasets published by the Government of India. The study also comprehensively included both meteorological and air pollution parameters and not either of them in isolation, unlike most of the current studies in the literature. To the best of our knowledge, this is the first study to investigate the relationship with the prevalence of acute onset AED and the effect of environmental factors and air pollution indicators in India. The authors have earlier described the trend analysis of epidemic keratoconjunctivitis and the effect of environmental factors on its prevalence in the same geography [17]. There are some limitations to the current study as air pollution is difficult to control and it is not possible to standardize the rate of exposure and the duration as well. The animal studies may not be extrapolated to humans as the concentrations may not be comparable, and the changes in the human eye may take a longer period of time, but nevertheless this lends insight into the same pathophysiology. Subsequent studies with larger data points and other pollution indices collected prospectively can further build on these initial findings. The objective of this study was to study the correlation between the timing of occurrence or exacerbation of AED and the environmental factors prevalent at that time. There could be a minor difference of the onset of symptoms and the presentation to the hospital in this large cohort of patients. We considered an acute exacerbation of less than 3 months of duration as recent onset and took the time of presentation of the patient to the hospital as the month of presentation, as the symptomatology would have been severe enough to seek care.

## 5. Conclusions

In conclusion, this study aimed to describe the correlation between the temporal pattern of presentation of recent onset AED with meteorological parameters such as environmental temperature, rainfall, humidity, windspeed, and the effect of pollution in a cohort of patients from the district of Hyderabad presenting to a multi-tier ophthalmology hospital network in India. The findings show that lower prevalence of AED cases was associated with an increase in rainfall and humidity, and a higher prevalence was associated with an increase of temperature and ground-level ozone during the year. Sensitization of the clinicians and patients alike on the temporal pattern of AED during the year and the effect of environmental factors may help care providers to develop a more holistic approach in treating these patients.

## Data Availability

Data sharing not applicable.
